# Atomistic Monte Carlo Simulation of Lipid Membranes

**DOI:** 10.3390/ijms15021767

**Published:** 2014-01-24

**Authors:** Daniel Wüstner, Heinz Sklenar

**Affiliations:** 1 Department of Biochemistry and Molecular Biology, University of Southern Denmark, Odense M DK-5230, Denmark; 2 Theoretical Biophysics Group, Max Delbrück Center for Molecular Medicine, Robert-Rössle-Str. 10, Berlin D-13125, Germany; E-Mail: sklenar@mdc-berlin.de

**Keywords:** Monte Carlo, phospholipid bilayer, cholesterol, diffusion, coordinate transformation, entropy, sampling

## Abstract

Biological membranes are complex assemblies of many different molecules of which analysis demands a variety of experimental and computational approaches. In this article, we explain challenges and advantages of atomistic Monte Carlo (MC) simulation of lipid membranes. We provide an introduction into the various move sets that are implemented in current MC methods for efficient conformational sampling of lipids and other molecules. In the second part, we demonstrate for a concrete example, how an atomistic local-move set can be implemented for MC simulations of phospholipid monomers and bilayer patches. We use our recently devised chain breakage/closure (CBC) local move set in the bond-/torsion angle space with the constant-bond-length approximation (CBLA) for the phospholipid dipalmitoylphosphatidylcholine (DPPC). We demonstrate rapid conformational equilibration for a single DPPC molecule, as assessed by calculation of molecular energies and entropies. We also show transition from a crystalline-like to a fluid DPPC bilayer by the CBC local-move MC method, as indicated by the electron density profile, head group orientation, area per lipid, and whole-lipid displacements. We discuss the potential of local-move MC methods in combination with molecular dynamics simulations, for example, for studying multi-component lipid membranes containing cholesterol.

## 1. Introduction

### 1.1. Membrane Simulations and the Time-Scaling Problem

Analysis of the molecular mechanisms underlying membrane lipid organization is of crucial importance for understanding the function of membranes in living cells. Recent interest has focused on the biophysical characterization of model membranes to explore the physical principles governing the behavior of biological membranes. For example, lipid phase behavior and separation was investigated in model membranes by fluorescence microscopy, neutron scattering and NMR, while cholesterol dependent lipid order in endoplasmic reticulum membranes was determined by EPR spectroscopy [1–5]. Despite a large increase in computational power, atomistic membrane simulations lack behind this experimental progress. This is largely due to the large time scale at which biologically relevant processes in membranes occur. While characteristic times for stretching of a C–C bond in a fatty acid tail are in the range of a few picoseconds, change of the lateral position of two lipids requires several nanoseconds, and transverse phospholipid migration in pure lipid bilayers takes place on a time scale of hours [6,7]. Similarly, important biological processes like vesicle formation during intracellular transport require the collective dynamics of hundreds of membrane components for several seconds. Molecular detail, however, cannot be ignored when developing models of these processes, since small structural changes in the involved lipids can promote or antagonize membrane budding and fusion [8,9]. In addition, biological membranes are of extreme complexity by consisting of several hundred different lipid species, transmembrane proteins, like G-protein coupled receptors and peripheral proteins like prenylated ras or the glycosylphosphatidylinositol- (GPI-) anchored folate receptor [10,11]. There is also the subcortical actin playing an important role in membrane organization and dynamics [12–17], the extracellular oriented glycocalyx [18] and the well-established transbilayer phospholipid asymmetry [19]. This compositional complexity together with the dynamically varying solute and ion composition on both sides of the plasma membrane naturally call for many different modeling approaches being suitable for each level of description [20–23].

### 1.2. Molecular Dynamics Simulation of Lipid Membranes

Atomistic molecular dynamics (MD) simulation, which solves explicitly Newton’s equations of motion, is now a very advanced and established technique being able to simulate the behavior of phospholipid membranes up to several hundred nanoseconds [7,24]. Important collective phenomena, such as thickness fluctuations and bilayer undulations, correlative lipid motion during diffusion, heat capacity changes due to increasing salt concentration, or cholesterol’s impact on the lateral pressure profile in lipid membranes have been studied by this approach [24–27]. Recently, cholesterol translocation has been studied by atomistic MD simulation using the replica exchange approach [28]. In this method, multiple replicas of the membrane are simulated in parallel at various temperatures to sample rare events, such as cholesterol flip-flop [28,29]. By this approach, the overall simulation time, including the advancement of the simulation clock for each detected rare event, could be extended tremendously to 15 μs altogether [28]. Local interaction of membrane proteins and lipids as well as the impact of fluorescent probes on bilayer properties has also been investigated by atomistic MD simulations [30–34]. MD simulations were combined with ab-initio calculation and spectroscopy experiments to determine dynamics of fluorescent dyes in ground and excited states [35]. Finally, using a specially designed supercomputer [36], classical atomistic MD simulations of protein-lipid systems were extended into the μsec-range and used to study voltage gating of a membrane embedded ion channel, to decipher conformational changes of the epidermal growth factor receptor on ligand binding and to characterize specific lipid interactions of receptor subunits [37,38].

Together, classical atomistic MD simulations provide information about many relevant membrane processes in the time window of several fs to <~0.5 μs for a few hundred lipid molecules. Using improved sampling schemes, such as replica exchange MD or supercomputer architectures optimized for MD simulations, the simulation time can be extended by a factor of ten. However, even with further improvement of computer instrumentation, it can be expected that other highly relevant but slower phenomena like lipid domain formation or phospholipid flip-flop in membranes taking place in the range of milliseconds to seconds and/or involving larger membrane areas cannot be studied solely by the MD technique. This is a disadvantage of the inherent time dependence of the method, which requires time propagation steps of typically 1–2 fs to follow the fastest processes, such as intramolecular vibrations. As a consequence, membrane phenomena occurring on a large time scale in nature will require lengths of MD simulation runs not accessible by today’s conventional computational power. Supercomputers, such as “Anton”, developed by David E. Shaw’s group, might provide a way to extend MD further towards the relevant mesoscopic scheme [36]. However, it is of large interest to develop alternative computational approaches reaching thermodynamic equilibrium of slow membrane processes within reasonable calculation time on conventional computer systems. One way is the coarse graining of the molecular lipid structure and force field, thereby allowing for efficient simulation of slow collective membrane phenomena, such as membrane domain formation, vesicle fusion, membrane crowding, or flip-flop of phospholipids and transbilayer coupling [39–41]. These advantages come to the price of neglecting molecular detail, such that fine differences in lipid structure, for example, between cholesterol and its various biosynthetic precursors [42], cannot be studied adequately by coarse-grained (CG) simulation. This is a rapidly growing field, and a detailed discussion of these developments is beyond the scope of this article. We will, though, briefly touch on this topic when we discuss the potential of local move Monte Carlo (MC) simulations for multiscale and CG modeling of lipid membranes in Section 5 of this article.

### 1.3. Monte Carlo Methods for Atomistic Simulation of Lipids and Other Biomolecules

As alternative to MD simulations of atomistic or CG molecular models, one can circumvent the explicit time dependence of the investigated processes and sample molecular conformations along a Markov chain in the configuration space based on their probability in a Boltzmann distribution [43]. This approach, which is at the core of the Metropolis MC method, samples different configurations from an ensemble of structures and should—According to the Ergodic theorem—Provide the same result as MD sampling of the time-evolution of a single structure [44]. Let *X*^N^ be the configuration vector comprising positional and rotational coordinates of an *N*-molecule system, *i.e.*, *X*^N^ = {*X*_1_, *X*_2_, ... *X*_N_}, average thermodynamic properties, such as the internal energy, U, of the system are expressed in a statistical sense as average according to:

(1) $$U=\int \cdots \int E(X^{N})\cdot P(X^{N})dX^{N}=\langle E(X^{N})\rangle$$

Here, E(*X*^N^) is the configuration energy, while the probability of finding the system in state *X*^N^ is given by

(2) $$P(X^{N})=\frac{\exp(-\beta \cdot E(X^{N}))}{\int \cdots \int \exp(-\beta \cdot E(X^{N}))\cdot dX^{N}}$$

This high-dimensional configuration integral and thereby the thermodynamic average properties, such as the internal energy, cannot be evaluated by numerical integration. However, P(*X*^N^) can be evaluated by generating an irreducible Markov chain, of which the limiting distribution is the Boltzmann distribution:

(3) $$P(X^{N})=\left\{P(X_{1}^{N}),P(X_{2}^{N}),\ldots \right\}$$

Here, $\text{P}(X_{i}^{N})$ is the Boltzmann probability of the *i*th configuration of the system; for a detailed discussion of the underlying statistical theory, see ref. [43–45]. The standard Metropolis MC method has been first applied to simulation of water and aqueous solutions of ions [46,47]. It was limited by slow convergence, *i.e.*, reaching the thermodynamic equilibrium of the system given by the limiting distribution of the Markov chain (see above) in reasonable computational time. This problem hampered the application of the method to larger molecular systems.

Early attempts to improve sampling efficiency modified the underlying transition matrix, thereby biasing the moves in a certain direction. For example, smart MC developed by Ross *et al*. included forces into the MC sampling procedure, thereby increasing the likelihood of moves in the direction of forces acting on the simple molecule models [48]. A similar modification of the Markov chain has been introduced by Pangali *et al*. in their force-biased MC algorithm [49]. Computationally faster equilibration of solvated rigid molecules has been also achieved in such early studies using a preferred sampling method, where solvent molecules close to the solute surface have been moved with higher frequency [50]. Not only intermolecular forces but also the virial in the *NPT*-ensemble can be included in the acceptance criterion to increase the sampling efficiency [51]. In addition, the step size of the attempted moves can be adjusted according to the total interaction energy of the particle [52]. Another way of accelerating equilibration of the system is to shift to the grand canonical ensemble (GCE) and to insert/delete molecules as part of the sampling procedure. Mezei and co-workers developed cavity-biased GCE simulations in which MC moves are only attempted into intermolecular cavities of suitable size [53]. In addition, other methods have been developed, and this short overview is not comprehensive, but just serves the aim of making the reader familiar with the major challenges in applying MC methods to molecular simulations. For a more general introduction into various MC methods and enhanced sampling techniques, the interested reader might consult the text book by Allen and Tildesley [44] or two recent reviews [54,55]. Similarly, the fruitful application of the MC method to two-dimensional lattice and off-lattice simulations of simple membrane models are covered elsewhere [22].

While being a problem in simulating particles in dense fluids, the low acceptance rate of standard Markov chain MC methods has been even a larger hurdle in MC simulations of non-rigid molecules and macromolecular assemblies, such as proteins or lipid bilayers. The reason for this is that intramolecular moves often result in steric clashes associated with high energy and thereby rejection of the attempted move. For example, even a modest change of a torsion angle in the middle of a molecular chain can lead to large movement of the atoms at the end of the chain causing, for example, clashes between neighboring fatty acyl chains of phospholipids in the membrane [56–58]. Several strategies have been developed to overcome this problem and to improve sampling efficiency. Reptation moves were proposed and applied, among others, to long chain n-alkanes and polymethylenes [59,60]. Here, an atom is removed at the end of one chain, while another atom is simultaneously added at the end of an adjacent chain. End rotation is the simple change of the last torsion angle in a chain altering the position of the last atom, only [61,62]. Another straightforward local MC move set is the flip move, in which an inner (united) atom of the chain is picked at random and rotated around the adjacent bond by an angle sampled from a uniform distribution in the range of 20°–30° [63]. This changes a total of four torsion and two bond angles. These three sampling methods replace only one atom per MC move, which is in contrast to MC moves, involving the geometric reconstruction of a group of atoms. One approach of the latter category is the so-called configurational biased MC (CBMC). In this method, an end segment of a chain molecule is removed and the missing part is rebuilt sequentially with a bias towards avoiding overlaps with the unmoved neighbor sites. Thus, CBMC introduce biases in the sampling procedure which sample certain torsion moves preferentially based on additional information about the studied molecular system [58,64]. CBMC has been combined with intervening MD simulations to enhance equilibration of a bilayer of 100 molecules of dipalmitoylphosphatidylcholine (DPPC) [57,65]. The cavity-biased GCE method has been also employed in conjunction with CBMC for simulation of a membrane patch of dimyristoylphosphatidylcholine (DMPC) resulting in improved sampling compared to MD runs of comparable length [66]. Permeation of small molecules across DMPC bilayers, as well as the effect of cholesterol on membrane permeability, has been studied by atomistic MC simulation in the torsion angle space with fixed bond length and bond angles [67–69]. Intramolecular rotations as well as whole lipid rotations were performed in an extension-biased scheme [67].

However, these approaches suffer from rather slow convergence, as small changes in torsion angles can be associated with large changes in configurational energy due to van der Waals contacts of distant atoms and steric clashes [70]. Much effort was, therefore, devoted to the development of local move sets with constant bond lengths and bond angle constraints for more efficient equilibrium ensemble simulations. The theoretical background of such work is provided by the classical paper of Go and Scheraga (1970) [71], where a general formalism for ring and chain closure in the space of dihedral angles (the “torsion angle space”) was developed. The basic idea of this type of local or “window” moves is to change first one torsion angle (the driver torsion) followed by adjustment of the next six dihedral angles in the chain in a way that keeps the remaining chain (*i.e.*, from torsion angle *ϕ* = 7 on) unchanged. This closure method is inspired by the inverse kinematic problem in robotics, where a robotic arm joint by several links must be placed in such a manner that a desired position of an end-effector is provided [72]. To ensure chain connectivity, the closure problem in torsion angle space is solved by sequential application of rotation matrices under the constraint of recreating the correct distance between the last atom of the moved fragment and the second fixed fragment of the chain [71,73–75]. The approach introduced by Go and Scheraga (1970) has been adapted to the use in MC simulations of chain molecules by the concerted rotation (CR) method, which ensured detailed balance by taking the different volume elements of the coordinate systems into account [76]. A number of special CR variants were developed and successfully tested in MC simulations of polypeptide structures [70,74,75,77,78] and lipid bilayers [79], and the CR algorithm was implemented as an option of the MC module for the CHARMM program [80]. Another solution to the local MC move problem was presented by Pant and Theodorou (1995) with their “end bridging” (EB) and “internal re-bridging” (IRB) moves [81]. As in the original CR method [76], the geometric problem of chain closure in torsion angle space was formulated as determining the coordinates of a moved trimer relative to two specially confined dimers [81]. In the EB method, the fixed dimers belong to two different chains, while in the IRB method the dimers belong to the same molecular chain. Thereby, the IRB method is equivalent to the CR move set, just with two driver torsions, one for each of the fixed segments. In both bridging moves, a set of nine equations is numerically solved, which links the Cartesian coordinates of the atoms of the bridging trimer to the constrained bond lengths. Here, bond lengths are defined between the connected adjacent atoms and as the distance between the second neighbors, essentially fixing the bond angles in the molecule [81]. The EB method alters the chain length distribution and is therefore suitable to simulate polydisperse polymer melts [81,82]. In these systems, proper weighting and simulation in the semigrand ensemble using EB, reptation, and end-rotation moves guarantee the correct polydispersity, *i.e.*, chain length distribution for the polymer model [81]. The EB method becomes more accurate for long chains, but cannot be used for lipid membranes, which is for the following reasons. First, EB moves alter the chain lengths, which is inappropriate for phospholipid bilayers, and, second, the performance of the EB moving scheme drops dramatically if the chains are ordered, as it happens to be in lipid membranes [62]. The bridging scheme of the original study [81] has been later extended for monodisperse systems using the double bridging moves [62]. It was also extended to internal segments of arbitrary lengths and combined with CBMC moves to systematically re-growth the internally moved segment [83,84].

Importantly, the torsion angle changes during a CR move are correlated such that the volume element in the Boltzmann factor exp(−*E*/*kT*)d*ϕ*_1_ ... d*ϕ*_6_ is not volume preserving. This necessitates inclusion of a Jacobian, *J*, in the Metropolis acceptance criterion as first shown by Dodd *et al*. [76]. The Jacobian accounts for the different volume elements in the Cartesian space *versus* the space of torsion angles and constraint variables. More generally, using any definition of generalized coordinates for describing the configuration space of a molecular system requires a transformation of the volume element in the configuration integral of the partition function. A subset of the new coordinates is usually considered as constraints (like bond lengths and angles in the CR procedure). Thus, the transformation of the volume element from the canonical Cartesian space into a new set of transformed coordinates reads:

(4) $$d\Gamma =\prod_{i=1,N}dx_{i}dy_{i}dz_{i}=J\prod_{i=1,M}dq_{i}\prod_{j=1,3N-M}dc_{j}$$

where {*q_i_*} is the subset of independent generalized coordinates, and {*c_i_*} are the constrained variables. The Jacobian *J* is the functional determinant of the transformation and is typically calculated as the inverse:

(5) $$J^{-1}=\left|\frac{\partial (q_{1},\cdots ,q_{M},c_{1},\cdots ,c_{3N-M})}{\partial (r_{1},\cdots ,r_{N})}\right|$$

Importance sampling, according to the Metropolis method, basically evaluates the integral over configurations of a subset of the configurational space in Cartesian coordinates. Therefore, the Jacobian has to be included in the Metropolis acceptance criterion to account for the coordinate transformation from cartesian to internal coordinates in the closure procedure. The Jacobian enters the acceptance criterion for the trial move *m* → *n* according to:

(6) $$p_{m\to n}^{acc}=\text{min}\left\{1,\frac{p_{n\to m}^{sel}\cdot e^{-E/kT}\cdot J(n)}{p_{m\to n}^{sel}\cdot e^{-E/kT}\cdot J(m)}\right\}$$

Here, $p_{n\to m}^{sel}$ and $p_{m\to n}^{acc}$ are the probabilities for selecting and accepting the move, respectively [56]. Omitting the Jacobians from the acceptance criterion results in wrong torsion angle distributions since it violates the criterion of microscopic reversibility of the moves. The latter is required to ensure detailed balance and thereby to sample the Boltzmann distribution correctly. All CR variants in the torsion angle space have in common that complex non-linear chain closure equations have to be solved, with at most 16 solutions in the general case. Hence, computationally demanding numerical procedures are involved that do not readily provide the full set of solutions. To overcome this problem, Mezei (2003) devised a method to choose that solution of the closure problem, which is closest to the original conformation before attempting the move. Making this choice eliminated all moves which are unlikely to proceed [79]. To maintain microscopic reversibility, it was tested whether the reverse rotation applied leads again to the original conformation, which was called the “reverse proximity criterion” [79].

Like the other CR methods, this approach is limited to moves in the torsion angle space, since bond lengths and angles are kept constant in the simulation. Fixing bond angles, however, is not in accordance with MD simulations and results in a rough, artificial energy landscape. For example fixing bond angles increases the energy barrier for a cis–trans conversion of alkanes significantly making this constraint less suitable for membrane simulations [78]. The problems encountered by using fixed bond angles in protein systems were first addressed by Bruccoleri and Karplus (1985) who modified the original CR method by allowing for limited bond angle variations [85]. Definition of a modified (*φ*,*ψ*)-torsion potential to compensate for otherwise increased torsional barriers also allowed for partially removing the problems caused by fixed bond angles [70]. These problems were finally solved by extending the CR approach to include flexible bond angles in the closure algorithm [78]. In the latter method called concerted rotations with angles (CRA), the mathematical formalism for solving the kinematic problem in torsion angle space was modified by replacing three torsions around dihedral angles with changing bond angles. The overall derivation of the closure equations is very similar to the classical CR method including the associated Jacobians. The pre-rotation, however, was additionally restricted by a Gaussian bias to ensure small structural changes likely being accepted during each move [78]. The algebraic expressions become simpler in the CRA method with only two branches in the polynomial equation for solving the closure problem compared to four branches in the original CR method. It was shown that the CRA local moving scheme results in faster equilibration and more soft energy landscape [78,86,87]. In addition to sequential rotations and rebridging, the chain closure problem has been formulated as a fixed-end move for polypeptide chains, in which a crankshaft rotation is performed between distant alpha carbons [88]. This local move type does also alter the bond angles adjacent to the respective alpha carbons and was found to be more efficient than the original CR method. Related crankshaft-type local move sets, in concert with extensive use of side chain structure libraries, have been repeatedly applied to conformational sampling of flexible loops in proteins, but to review this rapidly growing field in detail is beyond the scope of this article [89–91]. Very recently, the geometric problem of CR has been rephrased in such a way that an exact analytical solution for the chain closure could be derived instead of the tedious numerical procedure [92]. Having this analytical solution, the authors could express the coupling between pre- and postrotation as linear transformation allowing for construction of a probability distribution controlling all necessary degrees of freedom (DOF). More precisely, the interdependencies between the various DOF during the local move could be expressed as correlations in a multivariate Gaussian distribution. This information could be used during the move to avoid imbalances in angular variations of pre- and postrotations, a problem from which even the CRA method suffered [78,92]. The so-called “concerted rotations involving self-consistent proposals” (CRISP) move was combined with an implicit solvent description and was found to sample the conformational ensemble of ubiquitin and other proteins more efficiently than the CRA move set [92]. The CRISP move along with CRA, crankshaft, pivot, and other MC move sets has been implemented in a program suite called “Phaistos” for rapid conformational sampling of proteins in implicit solvents [93].

We have recently presented a very different local-move set MC method, the chain breakage/closure (CBC) algorithm, which solves closure equations in the bond/torsion angle space using the constant bond length approximation (CBLA) [94]. The CBC equations provide two simple analytical solutions to the closure problem. Jacobi factors were analytically derived for chain molecules as well as for the ribose ring of DNA molecules and included in the Metropolis acceptance criterion. The method was found to be computationally very efficient and highly suitable to simulate sequence-specific DNA structure and drug docking to DNA [95,96]. It set the stage for further important developments and application in the field of modeling nucleic acids [97,98]. To take advantage of this approach in MC simulations of phospholipid membranes, local move sets within the CBC framework were adapted and defined for describing structural variations of DPPC molecules. In the following, we describe how this can be used to simulate single DPPC molecules and DPPC membranes in an implicit solvent description. We give a detailed explanation of implementation of local MC moves within the CBC framework into lipid and membrane simulations. This will make the reader familiar with the technical details and the challenges of this type of molecular simulations. In particular, we describe the sequential moving of individual units in each DPPC molecule, the set up and details of the bilayer simulation and the analysis of the data. We demonstrate that local moves in the bond/torsion angle space can provide fast structural equilibration of DPPC molecules in an implicit solvent, and we describe several measures for assessing this equilibration. We also show the rapid, in terms of computation time, transition of a crystalline-like DPPC bilayer into the fluid state, and discuss the limitations of the used solvent description. We do not claim, however, that the presented data for the membrane simulation resemble comprehensive sampling of the conformational space of all the lipids in the bilayer, as this would require much more extensive simulations and a more elaborated implicit solvent model, both, clearly beyond the scope of this review article.

## 2. Simulation Details and Analysis of DPPC Structure as Monomer and in the Bilayer

### 2.1. Molecular Model and Assignment of Moved and Dependent Variables

**Figure 1.**
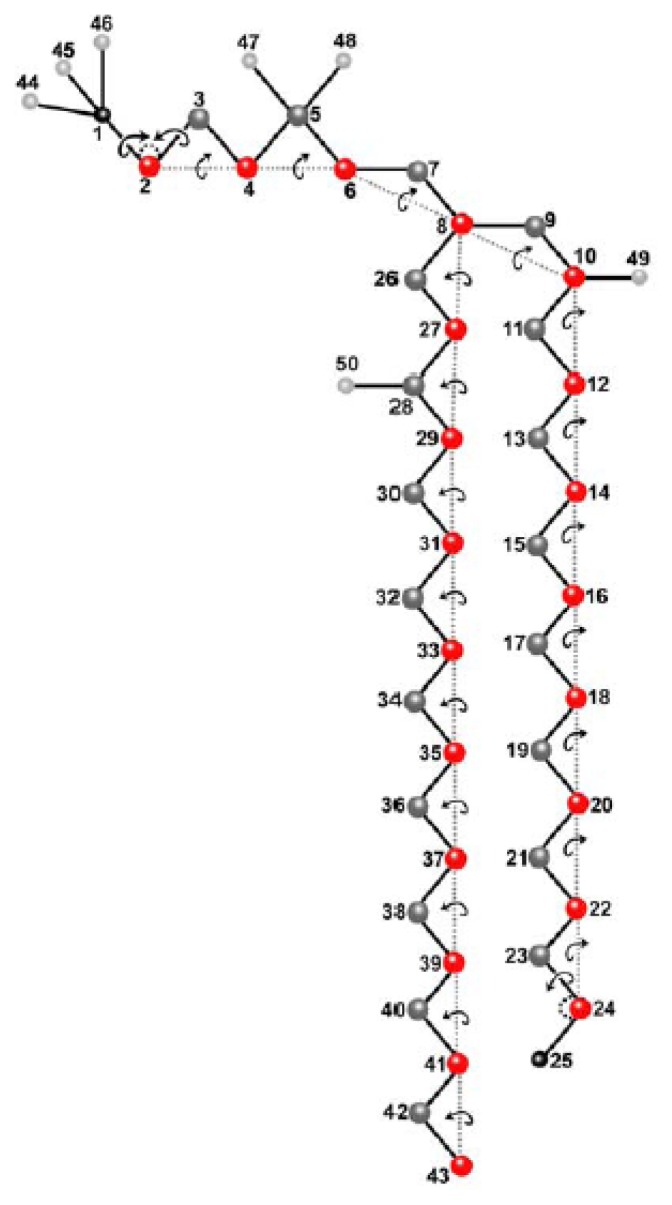
Molecular model of DPPC for atomistic Monte Carlo simulation. A united atom representation of dipalmitoylphosphatidylcholine (DPPC), which neglects hydrogen atoms, is employed reducing the number of atoms per DPPC molecule to 50 [81].

For some aspects of the lipid molecule, we used a united atom representation of DPPC recently developed for the AMBER 4.1. force field [99]. This DPPC parametrization combines methyl groups of the fatty acyl chains and methylen groups at the nitrogen and in the fatty acid tails, respectively, into one united atom, thereby reducing the total number of atoms from 120 to 50 per molecule (Figure 1). For the fatty acyl chains, the Ryckaert-Bellemann potential was applied as described [100,101]. A branched main chain of atoms in the DPPC molecule was defined leaving the united CH_3_ atoms (atoms # 44–46) on the headgroup nitrogen (atom # 1), the oxygen atoms (atoms # 47,48) on the headgroup phosphate (atom # 5), and the carbonyl atoms (atoms # 49,50) as external atoms (light grey in Figure 1, see preceding paragraph). From the 3*N* DOF, where *N* = 50 (number of (united) atoms) in the molecule, we have, due to CBLA and introduction of additional constraints, a reduction from 150 to 88 DOF. Figure 1 shows the local move sets defined for the variation of DPPC structures. The chain breaking fragments of the CBC algorithm are reduced to single atoms (red circles). After Cartesian moves of such atoms, the chemically bound neighbor atoms are repositioned by using the closure equations of CBC in the frame of CBLA. Crankshaft rotations of these atoms define additional DOF. Chemical moieties at chain ends or attached to the chain are moved by using classical internal coordinates (bond and torsion angles). All moves, including volume moves, were performed in a sequential manner including sequential updating of the energy. This has been shown to fulfil the balance condition and thereby to sample correctly from the Boltzmann distribution [102].

### 2.2. Start Configuration, Boundary Conditions, and Solvent Treatment

**Figure 2.**
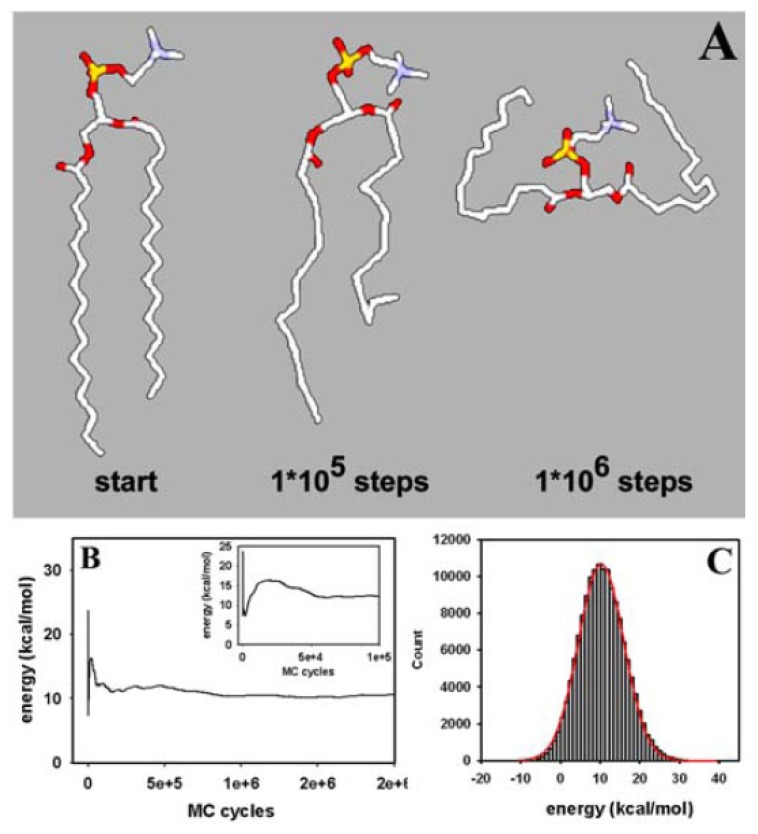
Snapshot and energy of a single-molecule Monte Carlo simulation. (**A**) starting conformation (start) and representative snapshots of a single molecule MC simulation are shown after 1·× 10^5^ and 1·× 10^6^ MC steps. Large conformational moves of the fatty acyl chains and the lipid head group can be observed; (**B**) mean system energy equivalent to the conformational energy calculated from the AMBER force field during the simulation [85]. The conformational energy becomes stable already after about 750,000 MC steps. The inset shows the initial phase of energy equilibration with a maximum after about 20,000 MC steps; (**C**) histogram of the conformational energy of the last one million MC steps (grey bars) overlaid with a fit to a Gaussian function of the form $f(E)=A\cdot \text{exp}\left({\left((E_{i}-\langle E\rangle )/\sqrt{2}\cdot \sigma \right)}^{2}\right)$ providing the mean energy at equilibrium, *E*, and the standard deviation, σ, as a measure of fluctuations around the mean value (red line). See text for further explanation.

A DPPC molecule was built using in-house modeling software. Values for bond angles, torsion angles, and bond lengths were taken from their equilibrium values defined in [99]. These values yielded straight fatty acyl chains of DPPC and a molecular conformation close to but not identical to the crystal structure previously defined for the closely related DMPC (PDB ID XP4; Figure 1) [103]. For one-lipid simulations, one MC cycle comprised changing all 88 DOF. Efficiency of energy calculations was guaranteed by calculating energy differences for each moved fragment with respect to all other atoms in the same lipid prior and after the local MC move. For the bilayer, the energy calculation included additionally the non-bonded interactions to other lipids (see below). The DPPC bilayer is approximately described by a two-dimensional periodic model with a cubic elementary cell, and the simulations were done under the condition of constant pressure of 1 atmosphere. This means that, in addition to structural moves, random variations of the volume have to be included, and the energetic contribution of the volume change adds to the structural intra- and intermolecular energy. The resulting enthalpy decides on the acceptance of the move by the Metropolis algorithm. We started with the crystalline-like conformation for 64 DPPC molecules (compare Figure 2A, “start” and Figure 5A). Cut-offs used in calculation of pair-wise (atom-atom) energies were defined by the minimum image convention [44]. For 6–12-Lennard Jones and electrostatic interactions a cut-off of 10 Å and a shell with a non-bonded list of 10 Å was generated. This list was updated every 10 cycles. That is, interaction of groups at a distance between 0 and 10 Å were calculated every cycle, while those of groups at a distance between 10 and 20 Å were stored in the pair list, similar as described previously [44,101]. Volume moves were attempted in every MC cycle for the bilayer by isotropic adjustment of the box length. For that purpose, the center-of-mass distance between all DPPC molecules was adjusted in each MC cycle. To account for effects of the solvent, an implicit description of dielectric properties of the water phase close to the bilayer/solvent interface was chosen. Thus, electrostatics was treated by employing a sigmoidal damping function for the dielectric constant *ɛ*(*r*) in the Coulomb term of the AMBER 4.1. force field function [104]. This is based on a function originally proposed by Hingerty *et al*. [105], and refitted by Lavery *et al*. [106], for large distances from the interface:

(7) $$ɛ(r)=d-\frac{d-1}{2}\cdot \left({\left(rs\right)}^{2}+2rs+2\right)\cdot e^{-rs}$$

where *d* is the plateau value and *s* the slope for the dielectric permeability. This function models the change of dielectric permittivity due to solvation of the phospholipid molecules. It, thereby, accounts for the overall effect of solvent (water) reorientation close to the solute surface. Simulations were run on an AMD PC with an Athlon XP 2700+ processor having 2.2 GHz clock frequency. On this machine, 10^5^ steps of MC simulation of a DPPC bilayer consisting of 64 molecules using the implicit solvent model required ten days of simulation time.

### 2.3. Evaluation of DPPC Conformations and Bilayer Structure

All trajectory analysis was performed using in-house developed Fortran77-based software. Systems energy and entropy were calculated from the trajectories of obtained conformations of the DPPC molecule. A histogram of molecule energies was generated for the one-lipid and bilayer simulation and was fitted to a simple Gaussian model using SigmaPlot 4.0 (SPSS Inc., Chicago, IL, USA). To calculate the entropy associated with the conformational ensemble of the single DPPC molecule, the following formula was used [107]:

(8) $$S=k_{b}\cdot \text{ln}(1+\frac{k_{b}\cdot T\cdot e^{2}}{\hbar }\cdot M⊙C)$$

Here, ***1*** is the unity matrix, ***M*** the mass matrix containing atomic masses in the diagonal, *k_b_* is the Boltzmann constant, *e* is Euler’s number, and ***C*** is the symmetrical covariance matrix whose elements ***σ****_ij_* are given by:

(9) $$\sigma_{ij}=\langle \left(x_{i}-\langle x_{i}\rangle )\cdot (x_{j}-\langle x_{j}\rangle \right)\rangle$$

**Figure 3.**
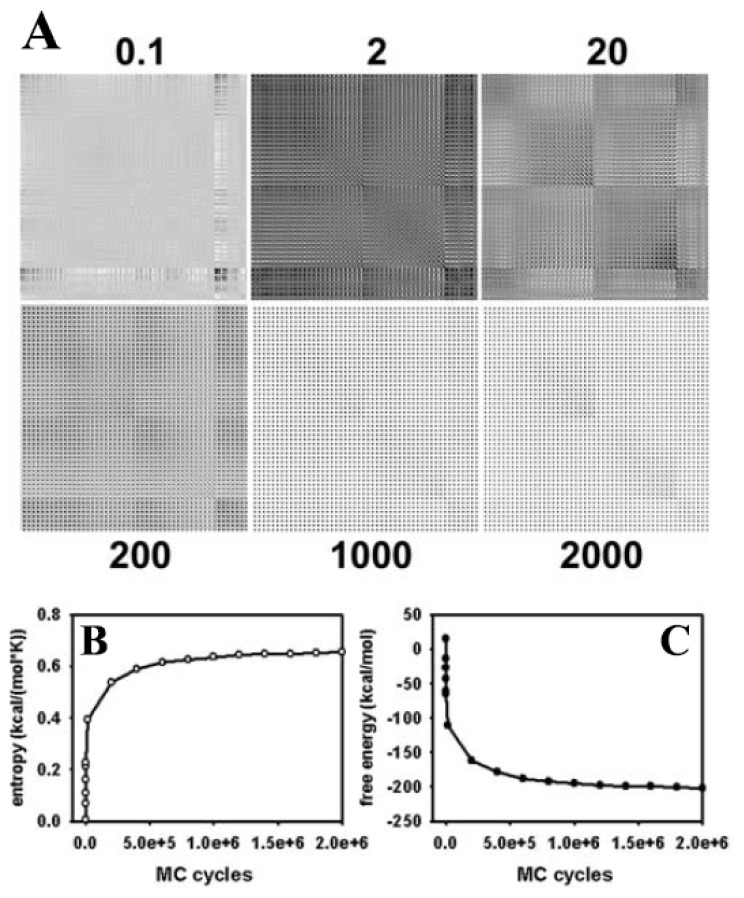
Covariance, conformational entropy and free energy of a single DPPC molecule. (**A**) from trajectories of single molecule simulations the covariance matrix of atomic positions was calculated and plotted as a 150 × 150 matrix for 100 (=0.1), 2,000 (“2”), 20,000 (“20”), 200,000 (“200”), one million (“1000”), and two million (“2000”) MC steps, respectively. Dark and light spots indicate high and low values of (co-)variances, respectively. The matrix values do not change grossly after 1 million MC steps with low off-diagonal values (*i.e.*, covariances). This indicates equilibration of the structural sampling and absence of significant correlations of fluctuations of adjacent atoms in the DPPC molecule; (**B**) the conformational entropy was calculated from the mass-weighted covariance matrix after a given number of MC steps, as described in the text; (**C**) the conformational free energy was calculated as *F* = *E* − *T*·*S* with *E* and *S* being the conformational (internal) energy and entropy, respectively. The temperature of the simulation was 323 K (*i.e.*, 50 °C).

The respective mean value in angular brackets is the average of all positions of an atom in Cartesian space calculated for a given number of MC steps. For example, the covariance matrix after 20,000 MC steps provides the variances and covariances of 20,000 conformations around the respective mean value separately calculated for each *x*-, *y*-, *z*-coordinate of the atoms in the DPPC molecule. Non-vanishing off-diagonal elements (covariances) indicate correlations between structural fluctuations of adjacent atom coordinates. The atomic Cartesian coordinates are given by *x*_1_, … *x*_3_*_N_*, and the matrix ***C*** has the dimension 150 × 150 for a single DPPC molecule. Values for the matrix ***C*** were calculated according to Equation (9), exported as text-file and plotted using the open-source image analysis software ImageJ (developed at the U.S. National Institutes of Health and available on the Internet at http://rsb.info.nih.gov/ij) in tagged image file (TIFF) format. The conformational entropy was calculated according to Equation (8), above, as function of MC steps (see Results section, Figure 3). The basic assumption here is that fluctuations are normal-distributed around the mean allowing for estimating covariances from a multidimensional Gaussian distribution [107–109]. The electron density profile was calculated by placing a Gaussian distribution of the electrons on each atomic center with the variance being equal to the van der Waals radius for each configuration and averaged over all configurations [110]. The P-N-vector was calculated as the angle between the P–N bond in the lipid head groups with the bilayer normal, and the distribution was calculated for all molecules in the bilayer and averaged over all configurations [99,111]. Head group torsions for the single lipid and the DPPC membrane were calculated using the same notation as described [99,103]. To assess the lateral diffusion of DPPC in the bilayer plane, the mean square displacement (MSD) was calculated according to:

(10) $$MSD=\frac{1}{N}\sum_{i=1}^{N}\sum_{s_{0}<S-s}{\left|\vec{r}(s_{0})-\vec{r}(s_{0}+s)\right|}^{2}$$

Here, *N* is the total number of lipids, *⇉* is the position vector of the center of mass of each lipid and *s* is the running index for the MC steps. An equivalent to a lateral diffusion coefficient, *D*, was estimated from the slope of the averaged *MSD* after an initial phase of box size adjustment in the constant *NPT*-ensemble simulation (see Results section) [27].

## 3. Results and Discussion

### 3.1. Efficient Monte Carlo Sampling of Conformations of a Single Lipid Molecule

**Figure 4.**
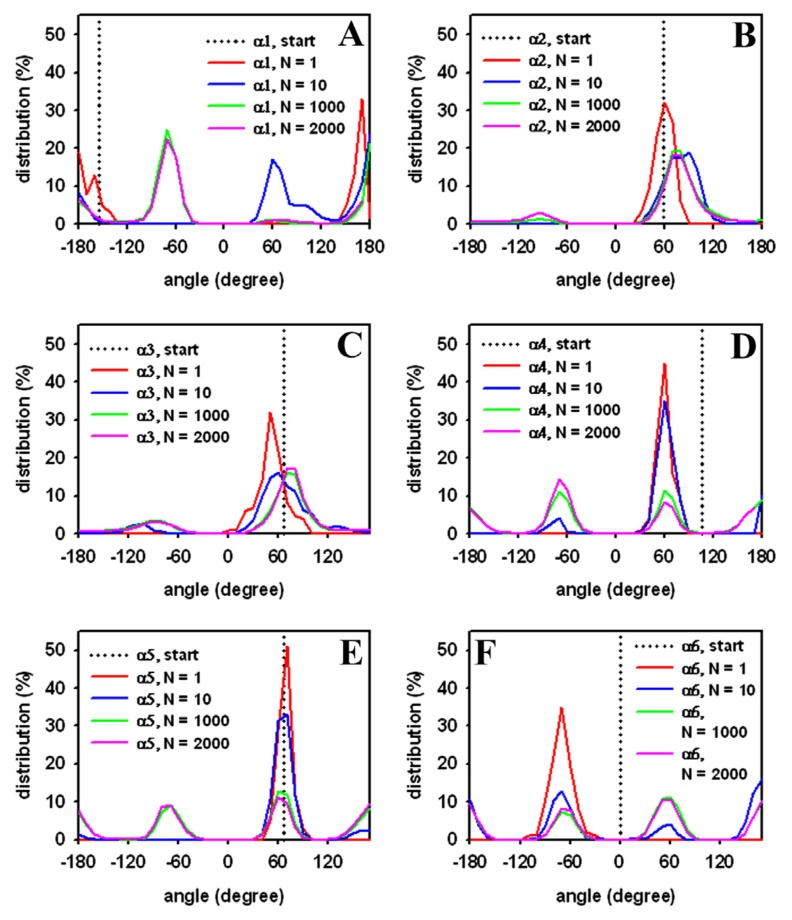
Distribution of DPPC head group dihedral angles from a single-molecule simulation. Head group torsions were defined as in Vanderkooi *et al*. [84] with the starting value defined by the initial conformation (dotted lines, “start”; compare Figure 2A). The percentage of occupation was calculated as function of torsion angle (in degree) after 1000 (“*N* = 1”, red line), 10,000 (“*N* = 10”, blue line), one million (“*N* = 1000”, green line), and two million (“*N* = 2000”, pink line) MC steps, respectively. The dihedral angles are defined as (**A**) torsions around atom 6 and 7 (α_1_); (**B**) atom 5 and 6 (α_2_); (**C**) atom 4 and 5 (α_3_); (**D**) atom 3 and 4 (α_4_); (**E**) atom 2 and 3 (α_5_); and (**F**) atom 1 and 2 (α_6_), respectively (compare Figure 2 for atom numbering in the DPPC molecule).

We started our simulations with a single DPPC molecule, as it allows us to assess structural equilibration of the basic unit of the membrane and to perform MC sampling of the whole conformational space. This is important, as thermodynamic properties calculated from bilayer simulations always average over individual lipids, which do not necessarily explore their configurational space individually. Thus, in order to assess equilibrium properties of the used molecular DPPC model, we performed first simulations of single DPPC molecules. This allows us to determine the evolution of structural correlations during MC sampling and thereby to assess the effect of the molecular constraints used in the description of DPPC (e.g., the CBLA and the move sets of external and closure atoms; see Figure 1 and Section 2.1., above). In addition, the properties of an equilibrated single DPPC structure can be directly compared with the same properties of DPPC in the bilayer assembly (see below and Figure 4 *vs*. Figure 6). Thereby, we can gather information about the degree of equilibration of the membrane during our simulation as well as about eventual structural confinement of DPPC in the bilayer. Both aspects are discussed below. Large structural moves in relatively short simulation times on a normal desktop computer were made possible by using the CBC moving scheme (Figure 2A). The conformational energy dropped, followed by a rise towards a local maximum before a stable value was reached after about 50,000 MC steps (Figure 2B). It can be concluded that the potential energy, *E*, for a single DPPC molecule is in the range of its equilibrium value after about 100,000 MC steps, while complete equilibration required about 750,000 steps. Fluctuations of *E* around the mean after obtaining a stable plateau value can be well approximated by a normal distribution (Figure 2C). This is a first indication that a thermodynamic equilibrium has been reached, since the probability density function (PDF) of the potential energy, *p_k_*(*E*), should be well approximated by a Gaussian function at equilibrium [112]. The PDF, *p_k_*(*E*), is the product of the density of states, *n*(*E*), a rapidly increasing function, and the Boltzmann weight factor (an exponentially decreasing function, as defined in Equations (2) and (3)) providing the bell-shaped energy distribution with maximum at the average conformational energy of the molecule at the given temperature [89,112]. From the non-linear regression one obtains an average conformational energy of 10.26 kcal/mol associated with a standard deviation as a measure for fluctuation of the conformational energy around the mean of SD = 5.71 kcal/mol. By squaring that value and multiplying with β *=* 1/(*k*_b_·*T*) with *T* = 323 K, we can determine the molar heat capacity of a single DPPC molecule in the canonical ensemble to *c*_V_ = 9.43 cal/mol·K [112]. For the united atom representation with 150 DOF and assuming for the sake of simplicity that the heat capacity ratio, γ, can be calculated like for an ideal gas (*i.e.*, γ = 1 + 2/DOF = 1.013~1), we would have approximately the same value for the heat capacity at constant pressure, *c*_p_. As we use the CBLA and thereby ignore stretching vibrations of the bonds, these values are presumably smaller than those obtainable by quantum mechanical calculations. However, it serves as starting point for comparison with the bilayer simulation (see below).

It is possible that the energy landscape for the lipid molecule is quite flat, meaning that a large number of different conformations are associated with very similar total energy. To obtain an independent proof for successful sampling of a representative subset of the conformational ensemble by our MC method, the conformational entropy was calculated. According to the Boltzmann theorem, the entropy is a measure of the probability of the sampled conformational subensemble and should, therefore, tend towards a maximum after long simulations leading to a thermodynamic equilibrium [44]. The conformational entropy can be calculated from the covariances of atomic positions, as outlined in Section 2.3, and Equations (8) and (9), above [107,108]. A stable plateau of the entropy would be equivalent to a covariance matrix with more or less constant values. In Figure 3A, the covariance matrix is plotted for all 50 atoms of the single DPPC molecule after various MC steps. It is apparent from the figure that values of the matrix do not change after one million MC steps, indicating that atomic fluctuations are in equilibrium. Some correlations persist in the equilibrated structure, as indicated by non-vanishing covariances after one and two million MC steps. This is likely a consequence of two facts; first, we have performed the calculation with Cartesian coordinates, which will naturally result in correlations between the *x*-, *y*-, *z*-directions for a given atomic position [113], and second, in the chosen geometry of the DPPC molecule, some groups are always moved in concert. This applies to the groups being defined as external atoms, such as the methyl groups at the nitrogen in the phosphatidylcholine head group or the carbonyl groups together with the fatty acyl chains (light grey spheres in Figure 1) and to the closure atoms belonging to each atom moved directly in Cartesian space (dark grey and red spheres in Figure 1, respectively) In addition, the local move MC set defined by the CBC equations naturally introduces correlations in the moves, which is the reason for the local and efficient sampling but also for the necessity of including the Jacobian into the acceptance criterion (see Equations (4)–(6) in Section 1.3. and [91]). Stable values of the covariance matrix after prolonged simulation is, thus, an important condition for ensuring reliable sampling of many independent conformations by the CBC algorithm.

The Schlitter entropy calculated from the mass-weighted covariances reaches a plateau after about 1,000,000 MC steps; *i.e.*, it needs longer simulations to equilibrate the conformational entropy than the energy, *E* (compare Figures 2B and 3B) [107]. From the entropy, *S*, and the conformational energy, *E*, of the molecule, the free energy, *F*, can be calculated according to *F* = *E* − *T*·*S*, where *T* is the temperature at which the simulation was performed [44]. As shown in Figure 3C, the conformational free energy reaches a plateau value after about one million MC steps indicating that the system has reached a thermodynamic equilibrium. The simulation time required for stabilization of the conformational free energy of a DPPC molecule is largely determined by the term *T·S*, *i.e.*, by the time for equilibration of the molecular entropy. The simulation time for obtaining constant values of the conformational entropy could be best described (χ^2^ = 0.9999) by a tri-exponential function with fractional half-times of 385 MC steps for the first phase, 11.179 MC steps for the second phase, and 266.595 MC steps for the third phase, respectively (not shown). The first and third phase contributed, each, to about 1/3 of the decrease in entropy, while the second phase contributed half of the entropy drop. Thus, the conformational entropy of our DPPC model is equilibrated by the CBC move set to about 83% of the stable plateau value with a half-time of less than 12,000 MC steps. The rather slow equilibration of the remaining one third of the entropy (the third phase of the fit) is not found for the evolution of conformational energy of the molecule, which became stable already after about 750,000 MC steps (compare Figures 2B and 3B). This suggests that many different conformations of DPPC are associated with a similar energy. Please note, that the absolute value of the entropy calculated from the covariance matrix is just an estimate based on the assumption that the PDF of atomic positions is a multivariate Gaussian distribution. Thus, the reached plateau values of entropy and, consequently, free energy might be different in case that non-Gaussian PDF’s underlie the fluctuation of atomic positions during the simulation. Together, calculation of conformational entropy and free energy, both reaching stable plateau values after about 1.5 × 10^6^ MC steps, indicates that our atomistic MC algorithm efficiently samples an ensemble of lipid conformations, thereby, faithfully, representing the configurational integral [44,55].

Next, the distribution of head group torsions was analyzed from a trajectory of MC simulated DPPC. The start value is given as a dotted line similar to a delta function, and only the alpha-torsions following the definition of Vanderkooi are shown (Figure 4) [103]. Already, after 1000 MC steps, a broad distribution of torsions angles close to a local torsion energy minimum is found for the DPPC molecule (red lines). This indicates large structural moves in a small number of steps, reflecting the sampling efficiency of the algorithm. After 10,000 MC steps, the second minimum starts to be occupied (blue lines), while, after one million steps, the local minima are occupied to a similar extent (green lines). No further change in these values was observed after two million MC steps (violet lines), suggesting that, after one million MC steps, the sampled ensemble represents the equilibrium state for a single DPPC molecule. Analysis of head group torsions therefore supports that the one-molecule system rapidly reaches a thermodynamic equilibrium when sampled by our MC algorithm. Note that this simulation takes only about ten minutes on a Pentium PC.

### 3.2. Large Conformational Moves Mediate the Transition from a Crystalline to a Fluid Bilayer

**Figure 5.**
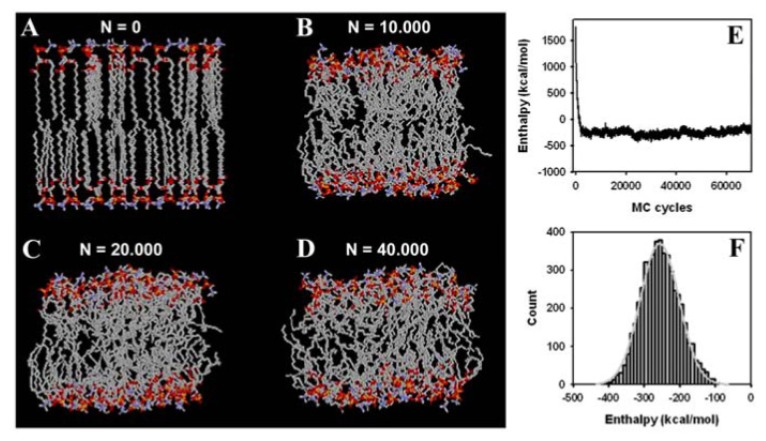
Simulation snapshot and system energy of a DPPC bilayer. The membrane simulation started from a crystalline bilayer consisting of 32 DPPC molecules with straight fatty acyl chains in each leaflet. Each molecule was rotated by a random rotation angle around the molecular long axis in the start configuration ((**A**), “*N* = 0”); (**B**–**D**) show snapshots after *N* = 10,000 (**B**); *N* = 20,000 (**C**); and *N* = 40,000 (**D**) MC steps, respectively. United atoms of methyl and methylen as well as carbon atoms are shown in grey, oxygen in red, nitrogen in blue, and phosphorus in yellow. Fatty acyl chains become increasingly disordered in course of the simulation; (**E**) the enthalpy of the bilayer in the implicit solvent was calculated after a given number of MC steps of the simulation performed in the constant *NPT*-ensemble. The temperature of the simulation was 323 K (*i.e.*, 50 °C); (**F**) a histogram of the conformational energy of the last 40,000 MC steps (grey bars) overlaid with a fit to a Gaussian function of the form $f(E)=A\cdot \text{exp}\left({\left((E_{i}-\langle E\rangle )/\sqrt{2}\cdot \sigma \right)}^{2}\right)$ provides the mean energy at equilibrium, *E*, and the standard deviation, σ, as a measure of fluctuations around the mean value (light grey line). See text for further explanation.

Phospholipids are naturally associated in membrane assemblies, and it is therefore important to prove that the MC algorithm faithfully simulates lipid membranes as well. Having shown rapid equilibration of an isolated DPPC molecule does not prove that densely packed DPPC molecules in a bilayer arrangement can be efficiently simulated. Our starting structure is a crystalline-like DPPC bilayer with straight fatty acyl chains and identical conformations for the 64 lipid molecules that comprise our system (see Section 2.2 and Figure 5A). We wanted to determine whether our local-move MC technique is able to produce conformational moves large enough to create a structural transition from a crystalline to a fluid-like state in the bilayer. As shown in Figure 5B, already, after 10,000 MC steps, the high molecular order typical for the crystal-like structure is lost and acyl chains of individual PC molecules show large conformational differences. Individual molecules are tightly packed, which is due to the fact that the box size in the *NPT*-ensemble simulation is rapidly adjusted (see also the accompanying video sequence). After 20,000 MC cycles, fatty acyl chains became more disordered (Figure 5C), while after 40,000 MC steps, the acyl chains of the phospholipids show large structural variation compared to the starting configuration. These changes indicate that the bilayer system made a transition to a fluid lipid bilayer. The simulated membrane model system is driven in a state of slight undulations, as suggested by the wave-like appearance of the head group regions (Figure 5D). This mesoscopic organisation has previously been described for long MD simulations of fluid DPPC membranes [25]. However, due to the simple solvent representation used in our MC simulation, we cannot rule out that these phenomena are caused or at least influenced by the simple solvent description. Starting from the crystalline-like ordered structure shown in Figure 5A, we determined next whether our MC algorithm leads to equilibration of the DPPC bilayer in terms of system enthalpy. As shown in Figure 5E, the system enthalpy drops to the equilibrium value in less than 10,000 MC steps, which are simulated in about one day on a Pentium PC. Thus, no energy minimization of the bilayer is required as often performed prior to extensive MD simulations (see [114] as example). A plateau value around −250 kcal/mol is obtained, which pertains stable during the simulation run. The PDF of the system enthalpy, *p_k_*(*H*), is well approximated by a Gaussian function with mean −256.7 and SD = 54.1 kcal/mol (Figure 5F) [112]. Using again the relation between fluctuation in energy (or enthalpy, as the membrane simulation was done in the constant *NPT*-ensemble; see section 2.2, above) and the heat capacity, we can derive a value of *c*_p_ = 821.63 cal/mol·K~0.822 kcal/mol·K. This can be used for a consistency check of one-lipid and bilayer simulation, as follows. Given the proportionality between heat capacity and system size [112], a value of *c*_p_ = 0.6 kcal/mol·K (*i.e.*, 64 times the value for the single lipid) would be expected. As the one-lipid simulation resembles individual DPPC molecules dissolved in an aqueous solution, one has to consider the additional heat capacity change due to transfer of lipid molecules from water to the hydrophobic membrane phase (*i.e.*, due to the hydrophobic effect [115]). This has been estimated to give Δ*c*_p_ ~ −0.1 kcal/mol·K per fatty acid chain of 16 carbon length [116]. Accordingly, based on this consideration and the one-lipid simulation, we would expect for the bilayer a value of *c*_p_ = 0.4 kcal/mol·K. Experimental values for the heat capacity of DPPC bilayers are in this range (e.g., ~0.15 kcal/mol measured by Heimburg and colleagues and ~0.3 kcal/mol as determined by Blume’s group, both using various forms of calorimetry [117,118]). Given the small bilayer patch, the simple implicit solvent representation, some uncertainty in the force field parameters and the used constrained bond lengths in our simulation, the value found here for the heat capacity is in satisfactory agreement with the experiment.

### 3.3. Rapid Transition of PC Headgroup Conformations by Local Move Set MC Sampling

**Figure 6.**
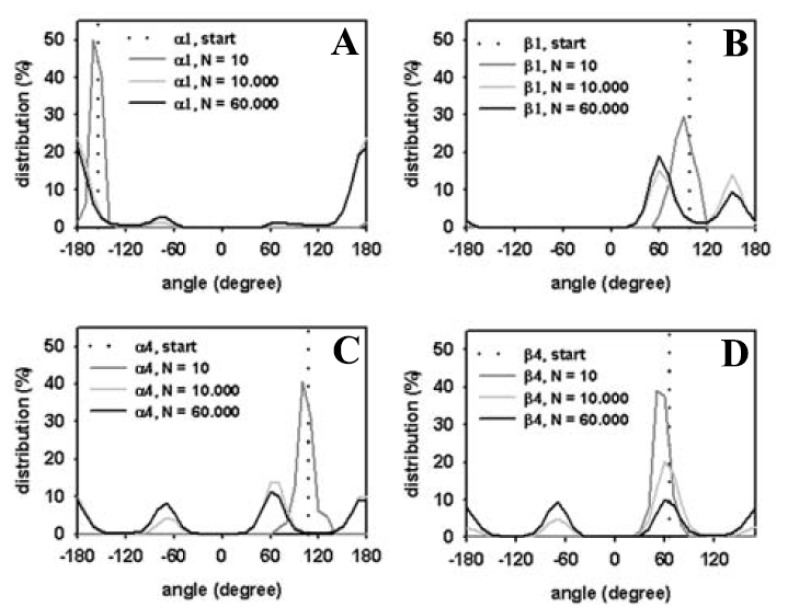
Head group torsions of DPPC in the membrane. Head group dihedral angles were defined as in Vanderkooi *et al*. [84], and as given in the legend to Figure 5. The starting value is defined by the initial conformation (dotted lines, “start”; compare Figure 2A). The percentage of occupation was calculated as function of torsion angle (in degree) after 10 (“*N* = 10”, dark grey line), 10,000 (“*N* = 10,000”, light grey line), 60,000 (“*N* = 60,000”, black line) MC steps, respectively. (**A**) shows the torsion around the bond connecting atoms 6 and 7 (α_1_); (**B**) around the bond connecting atoms 8 and 9 (β_1_) (**C**) around the bond connecting atoms 3 and 4 (α_4_) and (**D**) is the torsion angle distribution around the bond connecting atoms 11 and 12 (β_4_).

To quantify the extent of equilibration of DPPC headgroups during transition of the crystalline to the fluid bilayer state, we calculated the torsion angle distribution for selected head group torsion angles as performed for the single DPPC molecule (compare Figures 4 and 6). After only 10 steps, head group torsion angles occupy a relatively broad distribution around the starting value (dark grey line). After 10,000 MC steps, values for all torsion angles reveal a local energy minimum that starts to be occupied, while after 60,000 MC steps, energy minima of some head group torsions were equally occupied (see Figure 6C,D). By comparing, for example, the torsion angle distribution for the α4 torsion angle of phospholipids in the bilayer (Figure 6C) with that of the single DPPC molecule (Figure 4D) it becomes obvious that a similar level of occupancy is reached. Note, that we averaged over the given number of MC steps for all 64 phospholipid molecules in case of the bilayer, providing better statistics for a shorter simulation time than in case of a single DPPC molecule. In other words, the simulation of a single molecule for one million MC steps gives a very similar distribution for the α4 torsion angle in a DPPC bilayer with 64 molecules simulated for ~60,000 MC steps.

In contrast, the distribution of the *α*1 torsion angle clearly differs for the single molecule (Figure 4A) and the bilayer (Figure 6A). This torsion around the bond between atoms O(6) and C(7) in the lipid head group (the numbers in brackets are according to Figure 1) is therefore likely hindered in the bilayer compared to the free movement in a single molecule. Conformational equilibration for the bilayer lipids was also found for the β-torsions in the DPPC head groups after 60,000 MC steps (Figure 6B,D). We cannot rule out, however, that significantly longer simulation times would be required to ensure equilibration of all torsions in the head group region, especially those with high energy barriers due to steric confinement at the interface.

### 3.4. Electron Density Profile, Area per Lipid and Lateral Displacement of DPPC Molecules

**Figure 7.**
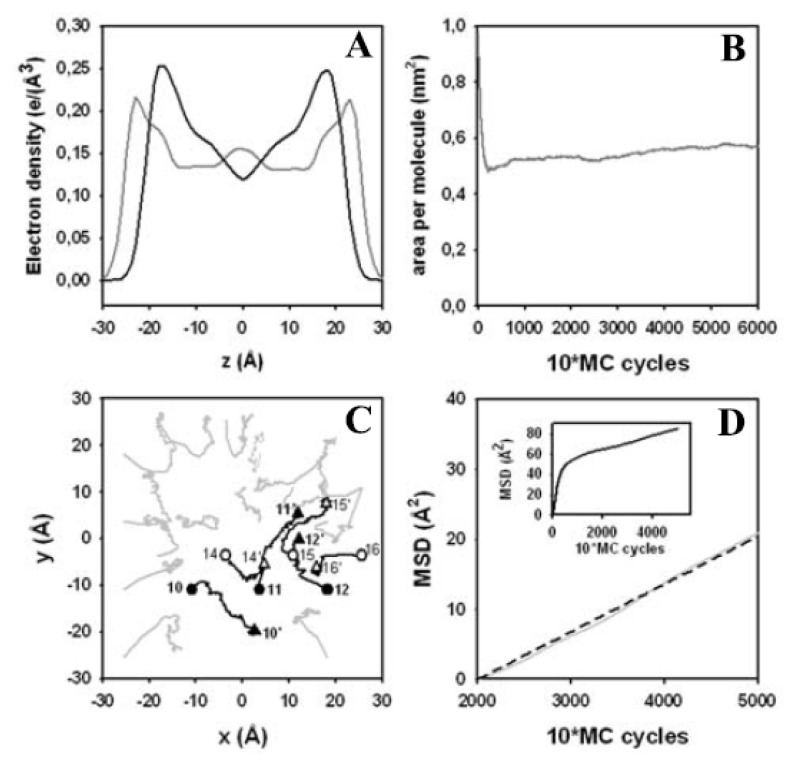
Electron density profile, area per lipid and lateral displacement in the bilayer. (**A**) the electron density profile was calculated for the starting configuration (grey line) and for the structure obtained after 60,000 MC steps (black line). One can clearly see that the membrane gets thinner with a large extent of fatty acyl chain disorder towards the bilayer center during the simulation; (**B**) the change of area per lipid from the crystalline start structure to the equilibrated value after 60,000 MC steps is shown; (**C**) a stroboscopic snapshot of selected lipid trajectories is shown for the upper leaflet during an MC simulation of 50,000 MC steps (lipid number X and X′ at the start, circles, and the end of the simulation, triangles). The position of the center of mass of the lipids is calculated. After adjustment of the box size causing initially large inward-directed “movement” of most lipids, the lipids show irregular “movement” as being characteristic for random walks (*i.e.*, diffusion); (**D**) the mean square displacement (MSD) was calculated between 20,000 and 50,000 MC steps. The MSD is linear (light grey line) and can be well described by a linear fit (dashed black line), as characteristic for normal diffusion (see text for values). Inset shows the MSD as function of MC steps (“10*MC cycles”) for the whole simulation.

Next, we evaluated the electron density profile parallel to the z-axis in the laboratory frame. In Figure 7A, the electron density profile of the starting structure is compared to that of the structure after 60,000 MC steps. The peak-to-peak distance in the head group region, *D*_HH_, a measure for bilayer thickness [6], is *D*_HH_ = 46 Å in the crystalline-like start structure and shows a characteristic double peak in the headgroup/glycerol region (grey line in Figure 7A). This double peak, as well as the value for *D*_HH_, is in line with X-ray diffraction data and earlier MD simulation of DPPC gel phase membranes [119,120]. Slight inter-digitations of the terminal part of the fatty acyl chains resulted in a small increase in electron density in this region. After 60,000 MC steps, the typical symmetrical density profile of a fluid DPPC bilayer with lowest electron density in the bilayer center was found (black line in Figure 7A). This confirms the transition from the crystalline-like to the fluid bilayer state during the simulation. The equilibrated bilayer structure is thinner (*D*_HH_ = 36 Å) than what is found in experiments for fluid DPPC bilayers at 323 K, *i.e.*, *D*_HH_ ~ 38 Å [6]. We assign this discrepancy to the simple solvent treatment in our simulation and to the united atom AMBER force field, which we used for DPPC. Interestingly, Smondyrev and Berkowitz, who developed this force field parameterization for DPPC [99], reported also a significantly lower peak-to-peak distance for DPPC in an MD simulation at 323 K with explicit water compared to the experimental value [99,121].

Another criterion for the transition from the crystalline-like start structure of the bilayer to a fluid membrane is the change of the area per lipid during the MC simulation (Figure 7B). After an initial sharp drop from 1.02 nm^2^ in the start structure to 0.53 nm^2^ after 2200 MC steps, the bilayer expanded in the following 40,000 MC steps to reach a plateau value of 0.6 nm^2^ area per lipid after 60,000 MC steps. Most of the initial drop is a consequence of the volume moves displacing whole lipids thereby resulting in tighter packing and a contraction of the simulation box. The subsequent increase in area per lipid is due to incremental mobility of the fatty acyl chains and their “melting” from the all-trans configuration [122,123]. In addition to intramolecular structural rearrangements during equilibration and conformational fluctuations at equilibrium, membrane phoshopholipids can exchange place by lateral diffusion in the bilayer plane. To determine whether our MC move scheme can cause significant lipid displacements in the membrane, the MSD of the center of mass of individual lipid molecules in the DPPC bilayer was calculated from the trajectory of an MC run and plotted as function of MC steps (Figure 7D). A stroboscopic snapshot of the center-of-mass position of tracked molecules in the upper membrane leaflet indicates that the DPPC molecules indeed perform large displacements during the simulation (Figure 7C). This confirms the fluid state of the DPPC bilayer reached after less than 30,000 MC steps. For example, lipid number 10 moves a distance of about 15 Å in the positive *x*-axis direction, while the lipid number 11 shows a similar displacement in y-axis direction. The MSD plot shows a steep increase for up to 10,000 MC steps, followed by a linear rise with much smaller slope after about 20,000 MC steps (Figure 7D, inset). While the initial rise again reflects primarily adjustment of the box size in our *NPT*-ensemble simulation as consequence of whole-lipid moves, the second linear phase indicates real Brownian-type diffusion. This part of the displacement curve can be fitted with a linear function (dashed line in Figure 7D). Following the Einstein relation for diffusion in a plane:

(11) MSD(t)=4·D·t

an apparent diffusion coefficient of *D* = 0.68 Å^2^/MC step can be calculated. We note that sampling along a Markov chain in configurational space, especially in internal coordinates, does not provide real dynamic information about the investigated molecular system [44]. The MSD is defined as the second moment, *i.e.*, the variance, of the PDF of the stochastic process underlying the observed time-dependent displacements. The PDF equals the step length distribution in tracking of randomly moving particles. Its time evolution is governed by the diffusion equation (*i.e.*, it is the propagator of the studied diffusion process). The MSD calculated here as a function of MC steps cannot be used to extract physically meaningful self-diffusion constants of DPPC or to test various membrane diffusion models. It is just used here to assess the efficiency local move MC simulations in “lateral scrambling” of lipids as precondition for studying lipid mixtures by atomistic simulation.

### 3.5. Impact of the Implicit Solvent on Area per Lipid and Head Group Conformation

**Figure 8.**
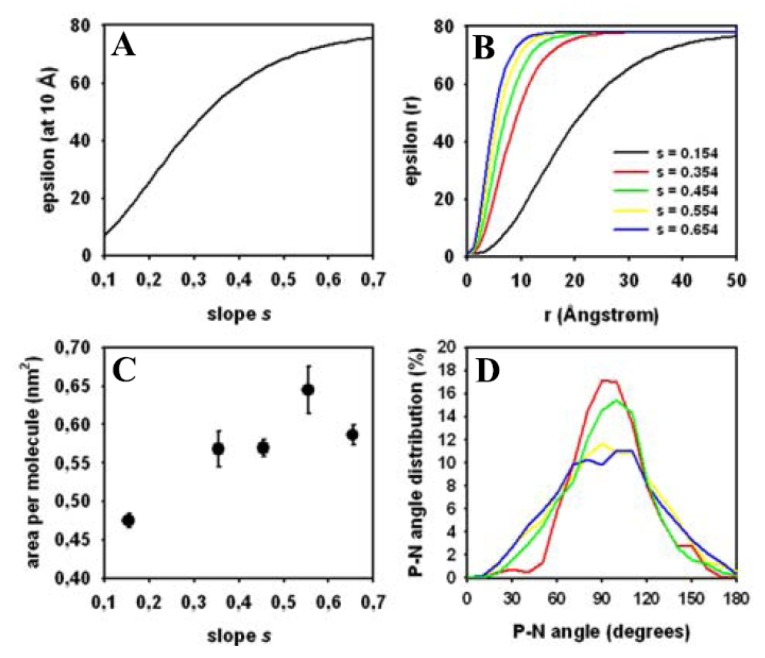
Impact of electrostatic interactions on membrane structure. (**A**) the distance-dependent dielectric constant is plotted as function of the slope, *s*, as defined in Equation (7) for a fixed distance from a charged group of 10 Å; (**B**) the distance-dependent dielectric constant is plotted as function of the distance from a charged group for various slope values of *s* = 0.154 (black line), *s* = 0.354 (red line), *s* = 0.454 (green line), *s* = 0.554 (yellow line), and *s* = 0.654 (blue line), respectively; (**C**) the area per lipid was calculated from the least 40,000 steps of separate MC runs performed with differing slope values of the dielectric constant. The mean ± SD is plotted as function of slope, *s*; (**D**) the head group orientation was inferred from the angle between the P–N vector and the bilayer normal and plotted for the separate MC runs. Increasing the slope, *s*, of the dielectric constant resulted in a broadening of the angle distribution (color coding is as for panel B). See text for more details.

The advantage in sampling efficiency of atomistic Monte Carlo simulations of biomolecular structures can only become significant if an implicit solvent description is used. This is because the computational cost of calculating solute-solvent interactions for explicit solvent surmounts the gain in sampling efficiency by MC compared with other methods [41,65,87,124]. As electrostatic interactions play a fundamental role in stabilizing bilayer structure [125], we next assessed the impact of the solvent treatment in our simulation. To this end, we systematically varied the slope, *s*, of the distance-dependent dielectric constant (see Equation (7)) and determined the area per lipid and the angle between the P–N vector and the bilayer normal from the trajectories of separately run MC simulation, each having 60,000 MC cycles (Figure 8). Varying the slope, *s*, determined at which distance from the bilayer surface the dielectric constant approached the value found for water (*i.e.*, ɛ = 78; Figure 8A,B). For example, for a slope, *s* = 0.65, the dielectric constant at a distance 10 Å from the membrane surface is ɛ = 74.69, which is close to the value in bulk water. In contrast, for a slope of *s* = 0.15, the dielectric constant at the same distance from the bilayer is only ɛ = 15.72, which resembles strong orientation of water molecules at the interface. There is a sigmoid dependence of ɛ as function of the slope, *s*, for a given distance from the surface (Figure 8A). We found that the area per lipid depends on the slope of the distance-dependent dielectric constant also in a sigmoid manner, with values of ≥0.6 nm^2^ for *s* > 0.5 (Figure 8C, mean ± SD). Thus, we chose a slope of *s* = 0.654 for all the simulations shown in Figures 5–7 to match experimental values as well as possible. The evolution of the area per lipid towards its equilibrium value was also found to depend on the slope value (not shown). The fluctuation around the mean value given by the respective SD of the area per lipid can be related to the bilayer compressibility modus *K*_A_ [123,126,127], for which we found in most simulations 260–430 mN/m. This is comparable to values found in equilibrated MD simulations [123,127].

The orientation of DPPC head groups was also affected by the parameter values chosen for the slope of the dielectric constant. For *s* = 0.154 and *s* = 0.354, the angle between P–N vector and bilayer normal showed a rather narrow peak at ~91° (Figure 8D, red and green curve, respectively). This constricted distribution is also found in simulations of DPPC bilayers in the gel phase [111], but also for certain lipid force field parameterizations [128]. It suggests that at low slope values of the dielectric constant the transition to the fluid bilayer state is incomplete in our simulations. For *s* = 0.554 and *s* = 0.654 we found a broad distribution of head group orientations with a maximum around 90°–100° (Figure 8D, yellow and blue curve, respectively). This has been similarly found in other MD simulations of DPPC membranes [99,128]. For *s* = 0.654 used in most of the simulations, there are two local maxima, one around 85° and one around 110°, while the overall distribution is rather broad (Figure 8D, blue curve). The maximum of the distribution suggests that DPPC head groups orient preferentially to cover the bilayer surface thereby efficiently shielding the hydrophobic acyl chains. In other words, this position of the P–N vector might have a function in covering the hydrophobic fatty acyl chains from contact to the aqueous environment [99,129]. Together, we found that the orientation of the P–N vector, similar as the bilayer thickness (see above), depend on the choice of the slope of the sigmoidal damping function in Equation (7) in Section 2.2. As this value is physically reasonable and produced the best agreement with experiments, we chose a value of *s* = 0.654 for this function in the simulation generating the results shown in Figures 5–7.

## 4. Summary and Future Improvements of the Presented Local MC Simulation Technique

In this study, we give a thorough introduction into atomistic MC simulations of lipid membranes. We describe the challenges and advantages of MC compared to other simulation methods and provide a concrete implementation of local move MC for efficient equilibration of lipid bilayer structures. While previous atomistic MC simulations of membranes use CBMC [65], cavity-biased moves [66], or local moves in the torsion angle space [67,79], our study shows the first implementation of an efficient move set in the torsion and bond angle space for lipid assemblies. As the CBC method allows large MC steps in the conformational space, it might overcome energetic barriers, which are not connected in a physical trajectory along phase space thereby enhancing the sampling efficiency. While this is true for other MC procedures as well, introducing flexible bond angles softens the energy landscape, which is particularly important for condensed systems of chain molecules, such as lipid bilayers. Some improvements of our method for future applications are as follows. First, we want to point out that the treatment of electrostatic interactions and the solvent description must be improved. Instead of the well known double-cut offs and pair lists for calculating the long-ranging interactions, particle-mesh Ewald or reaction field methods might be used [125,127]. A better implicit solvent model could be implemented based on the Generalized Born model, the ABSINTH implicit solvent description or the field integrated electrostatic approach (FIESTA) [87,91,124,130,131]. Special care has to be taken in implementing such models to account for the multibody interactions, which can conflict with the necessary decomposition of the energy function into static and changing terms for MC simulations [91]. In any case, explicit water should be avoided as it will diminish any advantage due to rapid sampling by lengthy calculations of solute-solvent interactions. In fact, we have tried initial MC simulations with explicit water based on the SPC2 water model and found that the computation time per MC cycle was, depending on the exact moving scheme, between six- to twelve-fold that of simulation with the implicit solvent description used here (not shown). A suitable compromise could be to use a mixed explicit/implicit solvent description. A physiologically relevant salt, such as NaCl could be added as co-solute and modeled explicitly, while the continuous description of water is kept. This has been done previously in local move MC simulations of DNA using the CBC algorithm [95] and would be highly relevant for membrane simulations, as salts like NaCl have been shown to affect phospholipid dynamics in membranes, both in experiments and simulations [27].

Second, refined force field parameters could be used likely giving bilayer structures in better agreement with experiments [132–134], which might be even more important than a more elaborated treatment of long-range electrostatics [128]. In fact, this issue touches upon the similarity between the united atom description used here for part of the DPPC molecule and CG descriptions of lipid structures. The empirically defined interaction potentials in CG models of lipid structure often include the solvent in the interaction parameters thereby also allowing for an implicit solvent description (see Section 5.2 below). In some of these CG models, a cohesive force between the fatty acyl chains is introduced to ensure stability of the bilayer state [135]; see below. Together, this suggests that better force field parameters might be obtained for a united atom description of the DPPC lipid structure by taking the aim of an implicit solvent description into account during force field refinement.

Third, local move MC simulation of lipid membranes might be improvable by appropriate parallelization of the program code. Due to the sequential moving and updating scheme, MC simulations are traditionally very difficult to parallelize. The only routine, which in principle should be easy to parallelize is the energy calculation, for the whole system as well as for the energy difference prior to and after attempted local moves. One way of parallelizing the whole MC run is by implementing the replica-exchange technique, also called parallel tempering, into the MC algorithm. This method is useful for sampling rare events in MD and MC simulations (see an example for membranes in the Introduction, Section 1.2). As long as the energy distributions of the system simulated at various temperatures overlap, and the system size is not too large, replica-exchange MC simulation can be very efficient for overcoming low-energy barriers [89,136]. Translated into a MC simulation of membranes, the procedure could comprise parallel local move MC simulation of replicas of the bilayer system at various temperatures distributed over several processors. As the simulation proceeds, configurations are exchanged between the systems at random and become accepted by a Metropolis-like criterion, if the high-temperature simulation happens to have found one of the low-energy regions of conformational space [29,55,89,136]. As an example of this, a massively parallelized replica-exchange MC simulation of a CG model of a polymer has been performed on graphical processing units (GPUs) with an almost linear relation between number of replicas and acceleration of the computation time for the system [137]. Alternatively, the MC move set can be directly parallelized by introducing a suitable decomposition scheme in which moves are attempted in parallel for regions of the simulated system separated at a distance larger than the longest inter-pair interaction. This decomposition could be the checkerboard arrangement of interaction sites, which has been first used on spin systems [138] and very recently in many particle simulations of hard disks on GPUs [139]. For local move MC simulations of chain molecules, Uhlherr *et al*. (2002) used a stripe decomposition, in which randomly chosen stripes across a simulated polyethylene molecule were defined as active [61]. The molecular model was a united atom representation of polyethylene, in which only short range Lennard Jones interactions and harmonic bond stretching were considered. In the active regions, various local move MC steps, such as CR, end bridging, *etc*. were performed in parallel. Suitable neighbor list were defined to update interacting united atoms, while any displacement attempts out of the currently active stripes were rejected [61]. Despite this progress, we believe that local move MC simulations of dense systems with many chain-like molecules, such as lipid bilayers, will be very challenging to carry out on several processors in parallel. Atoms belonging to different molecules will hardly be found in one active stripe only, as fatty chains in the bilayer interior are intersecting and closely packed. Accordingly, many attempted moves out of a stripe will be rejected. In addition, the necessary incorporation of long-range electrostatic interactions makes the definition of stripes with intervening non-active zones difficult, especially when Ewald summation techniques are used. The same challenges of including all necessary interactions are also found in parallelized MD simulations of membranes in which system decomposition is used. These problems have been satisfactorily solved in, for example, the Gromacs program suite [131,140], and might be possible to overcome also for local move MC simulations. In addition, for increasing membrane size, another simpler parallelization scheme useful for one bilayer system might be worth to consider: the costly energy calculation could be spread over several GPUs, while the Metropolis routine (*i.e.*, move, closure, and acceptance/rejection) could be run on the kernel-CPU. Indexing of interaction partners over an updatable pairlist would allow one to split the energy calculation efficiently over many processors/units.

## 5. Relationship between Atomistic MC Simulations and Other Membrane Modeling Techniques

### 5.1. Combination of Atomistic MC and MD Simulations

The last two decades have witnessed an enormous progress of biomolecular simulations, in particular in the field of modeling lipid membranes. In this area, simulations can be very fruitful, since in most experimental techniques only bulk bilayer properties are assessed. In contrast, molecular simulations of membranes can provide structural detail of the underlying lipid and protein dynamics. The simulations classically performed by atomistic MD, however, are very time-consuming and cover only membrane patches of a size of a few nanometer. This naturally calls for several developments: first, hybrid simulations using MD and other simulation approaches. We suggest that the local move MC method presented here is specifically useful in combination with MD simulations of lipid membranes. MD simulations provide dynamic structural information being directly comparable to experimental data. Assessing membrane dynamics with confidence requires proper equilibration of the simulated molecular system with its inherently broad range of time scales. Metropolis MC simulations of macromolecular systems, in particular in case of local and connectivity altering move sets, lack temporal information, as sampling occurs along a phase space trajectory [44]. Only for MC simulations of particles with very small step sizes and for croft CG representations of peptide chains with extremely small changes of torsion angles as the sole implemnented MC moves, a correspondence between MC steps and physical time could be established [141,142]. This is because the Metropolis MC sampling is a stochastic process, governed by a Markovian master equation, which allows for comparison with Langevin dynamics in these special cases [141]. On the other hand, connectivity altering local-move set MC methods, such as the CBC, CR, or CRA algorithms allow for overcoming physically disconnected energy barriers in the conformational space, which can be very useful for speeding up the equilibration phase of MD simulations. In fact, in previous attempts of combined MC and MD simulation of membranes, MD runs were interrupted with CBMC sampling to significantly enhance equilibration of a DPPC bilayer [65] and to improve diffusive mixing in two-component PC bilayers [143]. A direct comparison of combined CBMC/MD *versus* pure MD simulation for several single-type PC membranes found the first method three times faster in terms of CPU time for equilibration of the bilayers [144]. Chiu and colleagues used that approach additionally for pre-equilibration of sphingomyelin membranes, starting from a DPPC structure [145] and in two-component bilayers made of DPPC and cholesterol [146], for the CBMC moves, explicit water was removed, and the dielectric constant was set to one, which is even simpler than our implicit solvent description [65]. In addition, in these and similar studies [147], CBMC moves are only applied to enhance sampling of the phospholipids but not of cholesterol. This is not suitable for determining the lateral and transverse membrane distribution of this important lipid component. As the CBC approach is very general, it will allow for implementing the method to steroid-like molecules. Thus, we believe that local move MC algorithms, as the one shown here, hold great promise for rapid pre-equilibration of multi-component membranes, especially in combination with classical or advanced MD techniques [28]. This is also indicated in a mixed MC/MD simulation of a polyalanine peptide in which MD runs, based on the velocity-Verlet algorithm, were alternated with CRA moves explained in Section 1.3, above [148]. The authors found significantly faster formation of secondary structures compared to MD simulations alone. Efficiency of MC moves in such studies might be further increased by using force-biased MC techniques being introduced in Section 1.3, above. These techniques consume additional computational efforts by requiring calculation of forces which enter the Metropolis acceptance criterion. This disadvantage is eventually more than compensated by the possibility to define a reasonable MC time step, which—upon appropriate weighting—can be compared to the time step in the MD part of the simulation [90]. Timoneva *et al*. (2010) showed, based on earlier work, that a modified form of force-biased MC called uniform-acceptance force-biased MC (UFMC) allows for physically realistic simulation of diffusion and phase transitions in various materials [149]. In UFMC all displacements are accepted, which is in contrast to the original force-biased MC method by Pangali and Rao (1979) [49], but the moved distance is determined by the forces acting on the particle. These forces, in turn, follow a Boltzmann distribution, as long as they are sufficiently small which is guaranteed by predefining a maximal displacement [90,149]. A modification of this approach called time-stamped UFMC has been presented by Mees *et al*. (2012), where the maximal displacement of particle *i* was weighted by the square root of the mass of the lightest particle divided by the mass of the particle *i* [150]. This allowed for defining an average time step per simulation being directly comparable to the time step in MD simulations. Local move MC for biomacromolecular systems, as defined in the CBC and other algorithms outlined above (Section 1.3), can in principal also be extended towards variants of force-biased MC like UFMC. This, however, might be complicated by the complexity of the move operation in internal coordinates, making the definition of a meaningful time step challenging. Even if the latter would turn out to be prohibitively difficult, force-biased MC variants of the presented connectivity altering MC move sets should results in accelerated sampling and ease combination with MD simulations, potentially compensating for the time-consuming force calculation.

### 5.2. Atomistic MC Simulations in Multi-Scale Modeling of Lipid Membranes

A second development in the field concerns multi-scale modeling of lipid membranes. Coarse-graining of the molecular structure of various lipid species has been used to simulate membrane phenomena beyond the reach of classical all-atom simulations including lipid assembly and vesicle formation [41]. A particular challenge in this area is the development and validation of the molecular model, which allows for simulating lipid structures under various conditions, for example temperature-induced phase transitions. In addition to appropriate grouping of atoms with similar physical properties into defined “CG beads” [135], CG simulations require adjustment of the inter-particle interaction potential. There is no way of grouping atoms and defining interaction potentials from first principles. Thus, the interaction potentials between CG beads are necessarily effective potentials, which need to be inferred by matching lipid partition coefficients between oil and water [39] or by inverse statistical mechanics using data from all-atom MD simulations of the same system. A procedure using the latter approach is the inverse Monte Carlo method developed by Lyubartsev and Laaksonen (1995) [151]. This method is based on a theorem by Henderson (1974), which states that the interaction potential between atoms or molecules can be inferred from the radial distribution function (RDF) of the system at thermodynamic equilibrium [152]. Inverse MC starts with a distance-dependent discretization of the Hamiltonian of the system, which allows for relating the Hamiltonian to the RDF and the pair interaction potential [151]. In practice, inverse MC determines effective pair potentials by repeatedly performing MC simulations of a predefined CG molecular system, each time with slightly varied short-range interactions. These simulations provide estimates of the RDF for each variation of the interaction potential. As initial guess for the effective pair potential, the potential of mean force might be used. The RDF of this CG molecular system calculated for each MC run is compared to the RDF calculated for the same system in all-atom simulations, the latter being considered as ground truth. By minimizing the distance between the MC-estimated RDF and the “true” RDF, non-linear optimization, such as a Newton-Raphson method, allows for determining effective pair potentials. The procedure stops when the RDF of the CG system resembles that of the all-atom simulated system to a satisfactorily degree. The MC simulations during the optimization procedure consider only simple Cartesian moves of the CG beads in the CG model. Once the CG lipid model is established, it is used for simulations of large membrane systems which are beyond the reach of classical all-atom MD simulations. Such CG simulations are typically performed by MD or MC simulations, the latter with Cartesian moves of the CG beads, *i.e.*, the molecular sites [151,153]. We believe that local move MC simulations can be a very useful tool complementing the existing techniques for multiscale modeling of membranes in several ways. First, local move MC, for example in the CBC scheme developed here for the united atom representation of DPPC, can be applied to speed up the optimization procedure in inverse MC for obtaining the interaction potentials from the RDF (see above). Instead of simple Cartesian moves being prone for rejection due to steric clashes, the CBC moving scheme will allow for rapid exploration of the conformational space and consequently for reliable estimates of the RDF [151]. Second, local move MC might be applied directly to the simulations of the CG molecular model of a given phospholipid species. Indeed, in some implementations of CG membrane simulations, bond lengths are kept constant, while bond angle potentials between the CG beads are inferred from all-atom MD simulations [154]. Thus, connectivity altering local MC move sets, especially those which involve only three atoms per attempted move, as the CBC scheme discussed here, should be directly applicable to CG bilayer simulations and might result in faster equilibration compared to CG MD simulations.
